# Right ventricular involvement evaluated by cardiac magnetic resonance imaging predicts mortality in patients with light chain amyloidosis

**DOI:** 10.1007/s00380-017-1043-y

**Published:** 2017-08-24

**Authors:** Ke Wan, Jiayu Sun, Yuchi Han, Yong Luo, Hong Liu, Dan Yang, Wei Cheng, Qing Zhang, Zhi Zeng, Yucheng Chen

**Affiliations:** 1 Department of Cardiology, West China Hospital, Sichuan University, Chengdu, 610041 Sichuan People’s Republic of China; 2 Department of Radiology, West China Hospital, Sichuan University, Chengdu, 610041 Sichuan People’s Republic of China; 3 Cardiovascular Division, Department of Medicine, University of Pennsylvania, Philadelphia, PA USA

**Keywords:** Cardiac amyloidosis, Cardiovascular magnetic resonance, Survival, Right ventricular

## Abstract

Few studies have focused on right ventricular (RV) involvement in cardiac amyloidosis (CA). We investigated the prognostic value of RV assessment by cardiovascular magnetic resonance (CMR) in CA. In 2011–2014, consecutive patients with suspected CA referred for CMR were retrospectively evaluated. Demographic and baseline clinical characteristics were collected. Healthy volunteers were matched for sex and age and served as controls. All subjects underwent a contrast-enhanced CMR examination. RV size, function, and late gadolinium enhancement (LGE) were analyzed. All deaths during follow-up were recorded. Sixty-one patients [37 males (60.7%), age 60 ± 11 years] were included; CA was diagnosed in 47 (77.0%) patients. CA patients displayed decreased biventricular ejection fraction, elevated left ventricular mass index, and increased biventricular end-systolic volume index (ESVi) compared with controls. A total of 27 deaths (57.4%) occurred in the CA group at 21-month median follow-up. Multivariable analysis demonstrated that RVESVi (HR 1.033, 95% CI 1.004–1.063, *P* = 0.026) and RV-LGE (HR 2.814, 95% CI 1.063–7.450, *P* = 0.037) were independent predictors of mortality in CA. For all amyloid patients, log NT-proBNP (HR 3.412; 95% CI 1.484–7.845; *P* = 0.004) and RV-LGE (HR 4.149; 95% CI 1.623–10.607; *P* = 0.003) were identified as independent predictors. RVESVi and RV-LGE are independent predictors of survival and evaluation of RV by CMR enables risk stratification in patients with CA.

## Introduction

Amyloidosis is a group of rare diseases characterized by extracellular deposition of insoluble abnormal fibrillar proteins derived from various precursor proteins that leads to multi-organ structural alterations and functional impairment [1]. Cardiac involvement frequently occurs in immunoglobulin light chain amyloidosis (AL) and has a worst prognosis than all other pathogenic subtypes [2].

Most studies have emphasized the importance of left ventricular (LV) diastolic dysfunction in CA, but the value of the right ventricular (RV) function is usually ignored [3–6]. In the clinical setting, transthoracic echocardiography is widely available for the assessment of RV function. Some studies that evaluated RV systolic performance by Doppler myocardial imaging and speckle-tracking imaging have shown an association of RV systolic dysfunction with poor prognosis in CA [7–9].

Cardiovascular magnetic resonance (CMR) imaging is the reference modality for the quantification of RV volumes and systolic function [10]. Furthermore, CMR has emerged as a high specificity, noninvasive tool for the diagnosis of CA, with late gadolinium enhancement (LGE) reflecting the distribution of amyloid infiltration in the extracellular space [11]. It has been reported that the presence and extent of LV-LGE are useful predictors of adverse outcome in CA [12–14]. However, few data are available regarding RV involvement evaluated by CMR to predict prognosis in AL amyloidosis. Therefore, we aimed to investigate the impact of the RV parameters evaluated by CMR on all-cause mortality in AL amyloidosis patients.

## Materials and methods

**Fig. 1:**
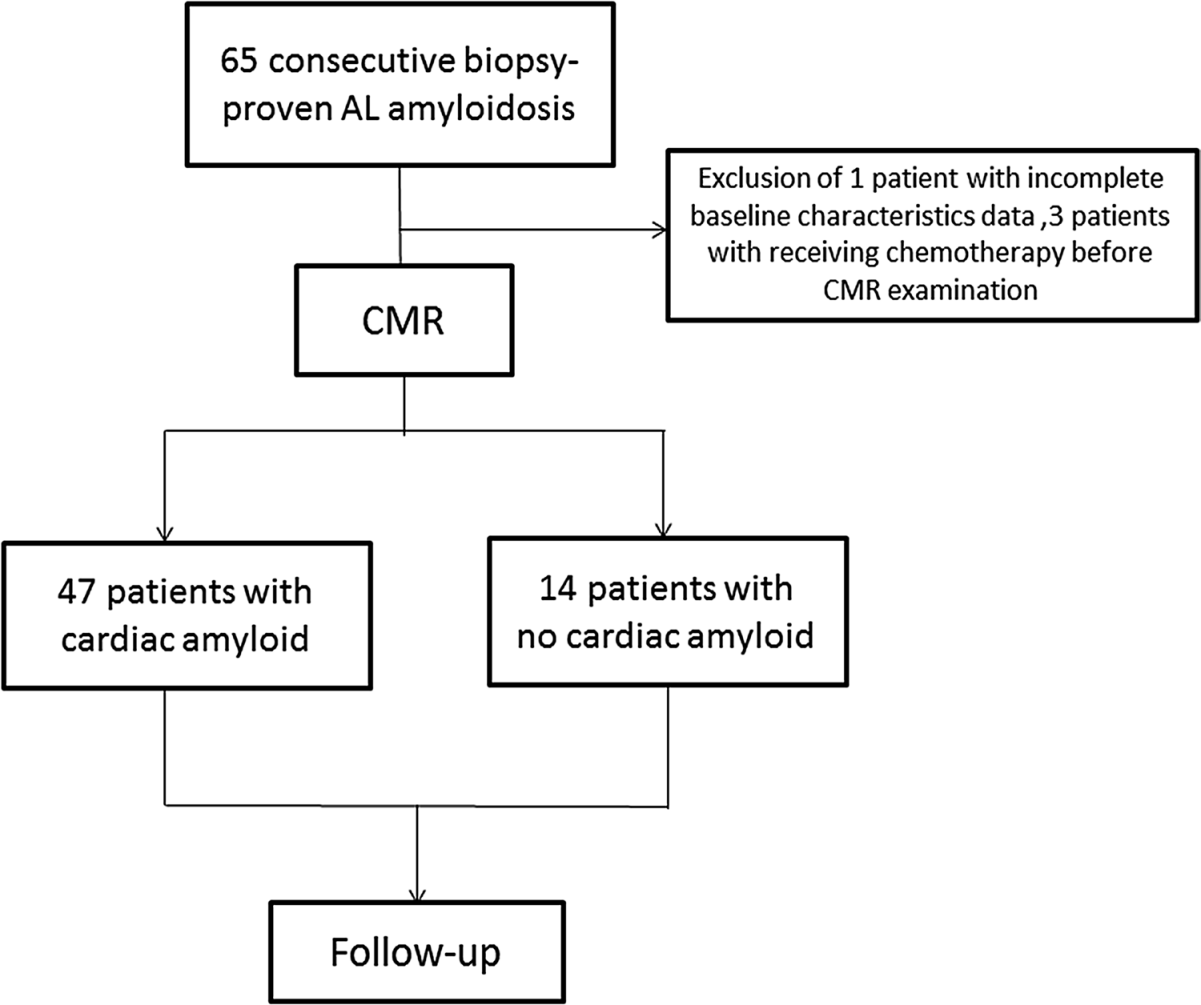
Patient selection flow diagram

Between 2011 and 2014, 65 consecutive patients with biopsy-proven AL amyloidosis in any organ system underwent CMR at West China Hospital, Sichuan University. In all cases, histological proof of systemic AL amyloidosis was obtained, with pink homogeneous material on hematoxylin and eosin staining, apple-green birefringence staining with Congo red, or amyloid fibers on electron microscopy. AL amyloidosis was further confirmed by immunohistochemical staining and demonstrating the presence of clonal plasma dyscrasias. Positive biopsy sites included abdominal fat (*n* = 21), kidney (*n* = 19), bone marrow (*n* = 14), lymph node (*n* = 3), bronchus (*n* = 2), rectum (*n* = 2), liver (*n* = 2), and skin (*n* = 2). Lack of cardiac involvement was defined as normal LV wall thickness without LGE on CMR and normal serum biomarkers. Three patients were excluded due to receiving chemotherapy before CMR, and one patient was excluded from the analysis due to incomplete data on baseline characteristics, resulting in a study population of 61 patients. Figure 1 shows the study’s flowchart. Fourteen patients with CA underwent chemotherapy after receiving CMR. Hematologic responses were evaluated according to the criteria of the International Society of Amyloidosis [15].

Forty-seven age- and gender-matched healthy controls were selected from our healthy volunteer database undergoing CMR with gadolinium. Healthy controls were enrolled based on responses to advertisements within the hospital and university and through personal contacts of the investigators. All healthy subjects had normal blood pressure (defined as <140/90 mmHg) and normal 12-lead electrocardiography (ECG) and showed no history or symptoms of cardiovascular disease or diabetes. All subjects included in the study provided written informed consent, and the study was approved by the Institutional Review Board of West China Hospital, Sichuan University.

ECG-gated CMR was performed during a breath-hold using a 3.0 T scanner (Magnetom Tim Trio; Siemens Medical Solutions, Erlangen, Germany) with an eight-channel phased array body coil. Steady-state free precession cine images were acquired in consecutive short-axis slices covering the whole ventricle and three long-axis slices (two, three, and four chamber views). The parameters of the SSFP cine were as follows: repetition time (TR) 3.4 ms, echo time (TE) 1.3 ms, flip angle 50°, field of view (FOV) 340 mm, matrix size 256 × 144, slice thickness 8 mm, no gap. LGE images were acquired at 3–5 and 10–15 min, respectively, after intravenous administration of 0.15 mmol/kg gadopentetate dimeglumine (Magnevist, Bayer Schering Pharma, Berlin, Germany) using an inversion recovery turbo-flash sequence in identical views [TR 700 ms, TE 1.56 ms, flip angle 20°, matrix 256 × 144, inversion time (TI) was individually optimized to null normal myocardial signal using a TI scout sequence].

Images were analyzed on a workstation using Q-MASS 7.6 software (Medis, Leiden, The Netherlands). Briefly, endocardial and epicardial borders were traced manually by an experienced observer in the end-diastolic and end-systolic frames on successive short-axis cine images. Ventricular volume was calculated by volume summation of consecutive short-axis slices. LVEF was calculated as: (volume end diastole − volume end systole)/volume end diastole). LV mass was derived by the summation of the disk method and by multiplying muscle volume by its density (1.05 g/cm^3^). The volumes of individual slices were summated to obtain the LV end-diastolic volume (LVEDV) and LV end-systolic volume (LVESV), which were then used to calculate LVEF [LVEF = 100 × (LVEDV − LVESV)/LVEDV] and LV mass (LV mass = myocardial LVEDV × myocardial density taken as 1.05 g/dL). LVEDV index (LVEDVi) and LVESV index (LVESVi) were defined as the ratio of LVEDV and LVESV, respectively, to body surface area. The papillary muscles were included in the LV volumes and excluded in the mass calculations.

In a similar manner, RV borders were identified on short-axis images at the end diastole and end systole. The interventricular septum was considered part of the LV, whereas the RV trabeculations were regarded as part of the RV cavity volume. On the basal slices, only the portion of the volume surrounded by the trabeculated myocardium was included in the RV volume, whereas the pulmonary valve and the right ventricular outflow track (surrounded by a thin and non-trabeculated wall) were not traced. From the identified borders, RV end-diastolic volume (RVEDV) and RV end-systolic volume (RVESV) were calculated and indexed to body surface area.

LGE images were first assessed visually for the presence and location of LGE by two experienced readers (D.Y. and H.L.) that were blinded to patient profiles and clinical outcome, with any disagreement adjudicated by a third expert reader (YCC). To exclude artifact, LGE was deemed present only if visible in two orthogonal views. Because the classification of cause of death is often problematic in AL amyloidosis, all-cause mortality due to progressive disease was the primary endpoint. Follow-up data were obtained by review of the patient’s hospital chart or telephone interview with the patient or relative. There was 100% follow-up.

Data were expressed as the mean + standard deviation (SD). Continuous variables were summarized as the mean ± SD where normally distributed with equal variances and compared by Student’s *t* test or analysis of variance. Differences among the three groups were assessed by one-way ANOVA or the Kruskal–Wallis test, followed by post hoc pairwise comparisons using Bonferroni correction. Nonparametrically distributed continuous data were presented as medians with interquartile range (IQR) and compared by the Wilcoxon rank sum test or Kruskal–Wallis test. NT-proBNP and troponin T were log (ln) transformed to achieve normality for further analysis. Variables for which *P* values in the univariate analysis were smaller than 0.05 were entered in a multivariate Cox proportional hazard model. Factors independently associated with the primary endpoint were identified using backward stepwise selection. We tested four models. The first model included the significant clinical variables, the second model included all significant biological variables, the third model included the significant functional and tissue characteristics of the CMR, and the last model included all significant clinical, biological, and CMR variables. Area under the curve (AUC) of the receiver operating characteristic (ROC) analysis was performed to determine the prognostic value and optimal cut-off point. Sensitivity, specificity, positive predicted value (PPV), and negative predicted value (NPV) using relevant cut-offs were computed. Survival curves were plotted with the Kaplan–Meier method using the log rank test for comparisons. All statistical analyses were performed using SPSS 17.0 software for Windows (IBM, Armonk, NY, USA). *P* < 0.05 was considered statistically significant.

## Results

### Study population

Sixty-one patients with AL amyloidosis [37 male (60.7%), age 60 ± 11 years] were included in the study; CA was diagnosed in 47 (77.0%) patients. The CA patients’ mean age was 60 years (IQR 54–68), 28 (59.6%) were men, and six (12.7%) had atrial fibrillation. The baseline demographic and clinical characteristics of the patients and healthy volunteers are summarized in Table 1. Compared with patients without CA and healthy subjects, patients with CA had a lower body mass index (BMI), worse biventricular function, and higher left ventricular mass index (LVMI). Biventricular end-systolic volume indices were higher in patients with CA compared with patients without CA, but diastolic volume indices were similar.

**Table 1:** Baseline demographic and cardiac magnetic resonance imaging parameters

	Healthy controls (*n* = 47)	Patients		*P* value
		No cardiac amyloid (*n* = 14)	Cardiac amyloid (*n* = 47)	
Age (years)	60 ± 11	57 ± 13	60 ± 11	0.525
Male/female	28/19	9/5	28/19	0.945
BMI (kg/m^2^)	23 ± 3	24 ± 2	21 ± 4^†,‡^	0.002
LVEF (%)	67 ± 5	60 ± 10^†^	47 ± 12^†,‡^	<0.001
LVEDVi (mL/m^2^)	72 ± 12	72 ± 15	73 ± 19	0.959
LVESVi (mL/m^2^)	24 ± 7	30 ± 11	39 ± 14^†,‡^	<0.001
LVMI	51 ± 9	66 ± 13^†^	92 ± 31^†,‡^	<0.001
RVEF (%)	61 ± 5	55 ± 7	45 ± 15^†,‡^	<0.001
RVEDVi (mL/m^2^)	69 ± 15	70 ± 17	64 ± 19	0.391
RVESVi (mL/m^2^)	27 ± 7	27 ± 5	35 ± 14^†,‡^	<0.001

*RVEDVi* right ventricular end-diastolic volume index, *RVESVi* right ventricular end-systolic volume index, *RVEF* right ventricular ejection fraction, *LVEDVi* left ventricular end-diastolic volume index, *LVESVi* left ventricular end-systolic volume index, *LVEF* left ventricular ejection fraction, *LVMI* left ventricular mass index

^†^ *P* < 0.05, compared with healthy subjects

^‡^ *P* < 0.05, compared with patients without cardiac amyloid

Survivors and non-survivors at 6 months follow-up did not differ in age, sex, low voltage, or chemotherapy (Table 2). Fourteen patients with AL amyloidosis underwent chemotherapy after CMR; the most common treatments included corticosteroids (100%), lenalidomide (64.2%), cyclophosphamide (50%), and bortezomib (21.4%). Five patients demonstrated a partial or better response to chemotherapy and three patients suffered progression in the survivor cohort; four patients had a partial or better response and two patients suffered progression in the non-survivor cohort. Hematologic response was similar between the survivor and the non-survivor cohort. In the CA group, 25 patients exhibited low voltage on ECG, 16 were in NYHA class III, and nine were in NYHA class IV. NYHA class was higher in patients who died than in patients who survived. Patients who died within 6 months showed higher NT-proBNP levels than those who survived. CMR imaging data are shown in Table 2. Patients who died within 6 months displayed greater RVESVi, lower RVEF, and similar RVEDVi compared with those who survived within 6 months. The RVESVi of the healthy volunteers was 27 ± 7 mL/m^2^. LV-LGE and RV-LGE were less frequently observed in the survivor group.

**Table 2:** Comparison of clinical and CMR parameters between 6-month survivors and non-survivors with cardiac amyloidosis

Characteristic	Survivors (*n* = 30)	Non-survivors (*n* = 17)	*P* value
Demographics			
Age (years)	61 ± 10	59 ± 11	0.462
Male [*n* (%)]	19 (63.3%)	9 (59.2)	0.255
NYHA class	2.0 (1.0–3.0)	3.0 (2.0–4.0)	0.026
Low voltage [*n* (%)]	14 (46.7%)	11 (64.7%)	0.362
Chemotherapy [*n* (%)]	11 (36.7%)	3 (17.6%)	0.204
Biochemical biomarkers			
*κ*:*λ* ratio	1.5 ± 1.0	1.4 ± 0.7	0.678
Troponin T (ng/L)	49.1 (25.5–116.4)	61.0 (61.0–209.4)	0.023
NT-proBNP (pg/ml)	4087 (1534–7872)	8338 (3820–14248)	0.058
Creatinine (μmol/L)	73.9 (52.9–104.4)	94.9 (69.4–136.0)	0.022
AST (IU/L)	25.0 (20.0–37.0)	34.5 (26.3–44.0)	0.031
ALT (IU/L)	22.0 (13.0-35.0)	25.0 (21.0–37.0)	0.227
UA (μmol/L)	393 (354.5–495.3)	485.5 (408.5–617.3)	0.062
CMR volume			
LVEF (%)	48 ± 12	44 ± 11	0.186
LVEDVi (mL/m^2^)	74 ± 20	71 ± 17	0.622
LVESVi (mL/m^2^)	38 ± 15	40 ± 13	0.654
LVMI	85 ± 31	105 ± 27	0.031
RVEF (%)	48 ± 14	38 ± 13	0.021
RVEDVi (mL/m^2^)	61 ± 19	69 ± 20	0.201
RVESVi (mL/m^2^)	31 ± 12	43 ± 15	0.008
LV-LGE [*n* (%)]	22 (73.3%)	17 (100%)	0.038
RV-LGE [*n* (%)]	13 (43.3%)	14 (82.4%)	0.015

*AST* aspartate aminotransferase, *ALT* alanine aminotransferase, *UA* uric acid, *LGE* late gadolinium enhancement

### Univariate and multivariate analyses in AL patients with cardiac amyloidosis

**Fig. 2:**
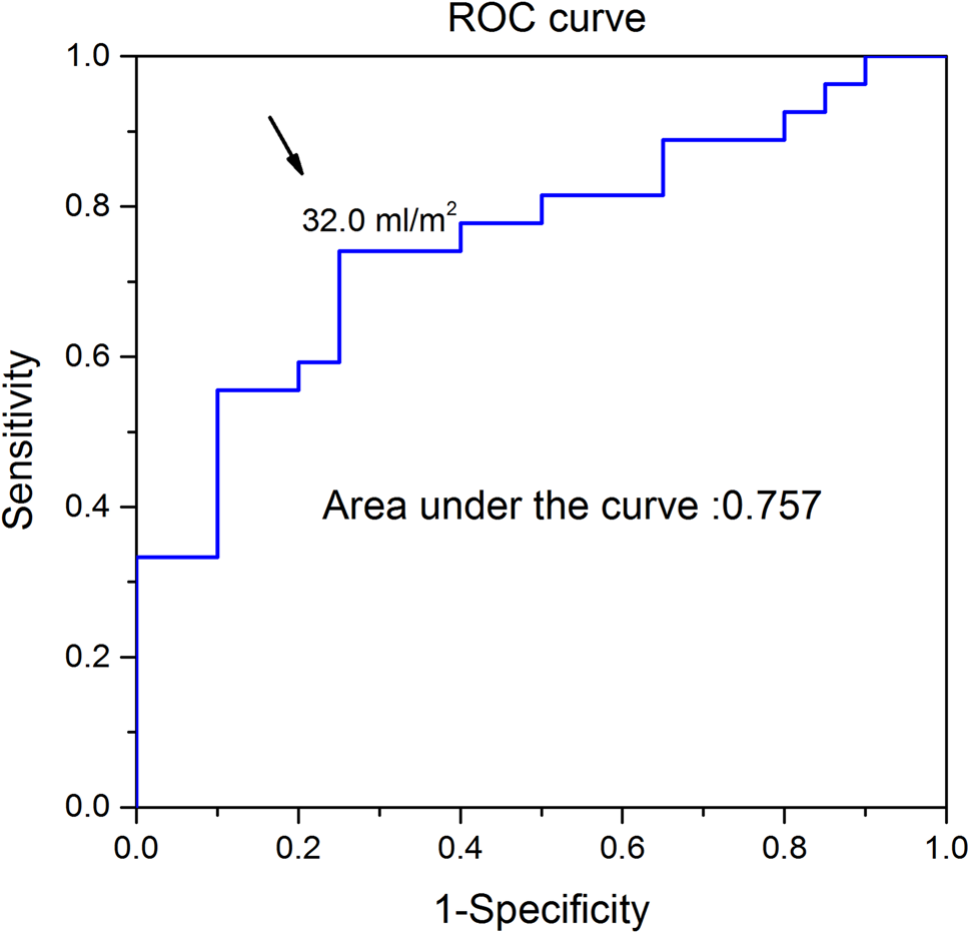
ROC analysis of the study population indicated that an RVESVi of 32 mL/m^2^ was the most suitable cut-off value for predicting death from all causes

**Fig. 3:**
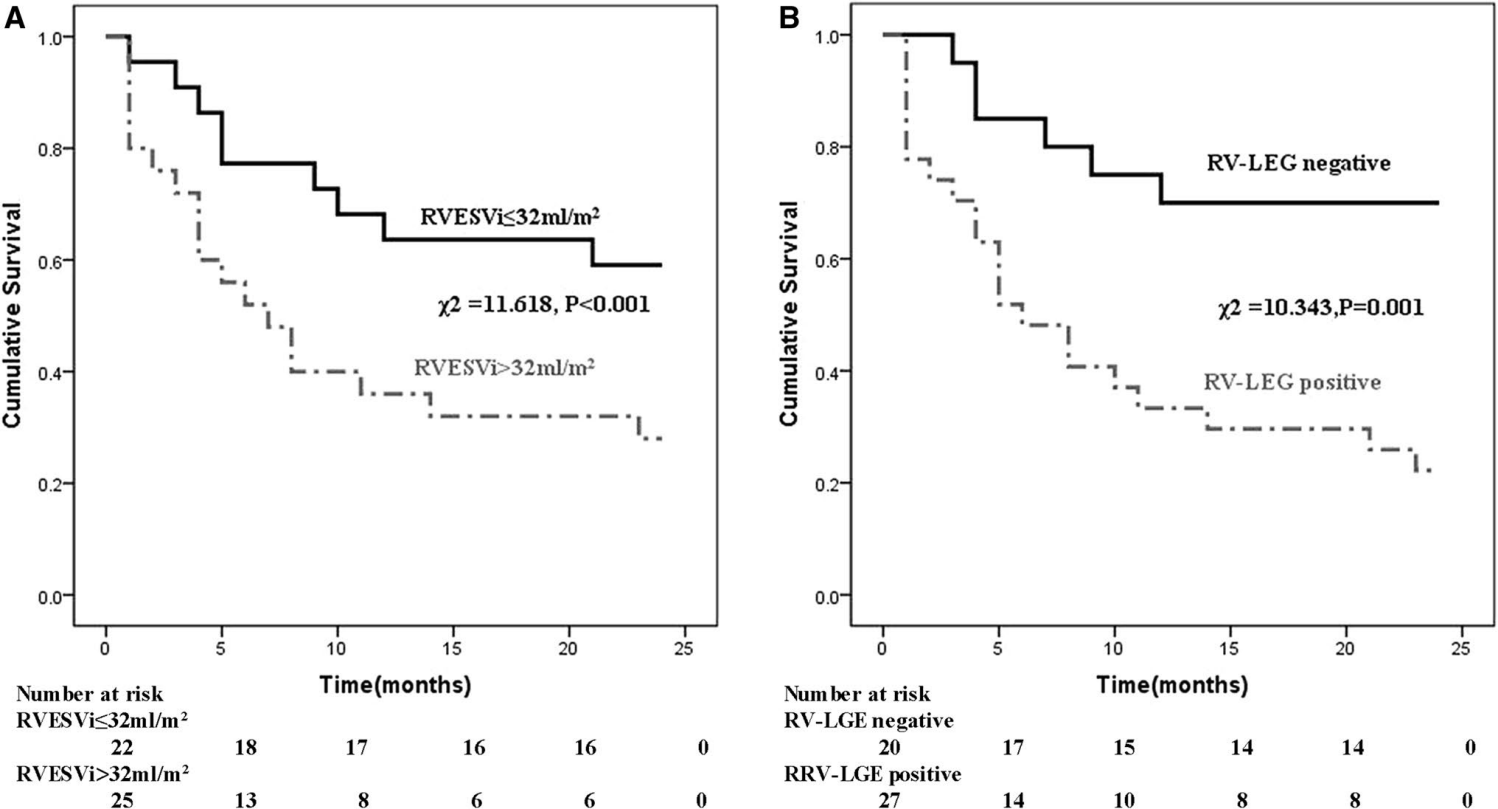
Complication-free survival curves by Kaplan–Meier analysis. **a** ROC curve-derived cut-off value of RVESVi of 32 mL/m^2^ predicted death. **b** Significant differences were observed in patients with positive RV-LGE compared with patients who were RV-LGE negative

**Fig. 4:**
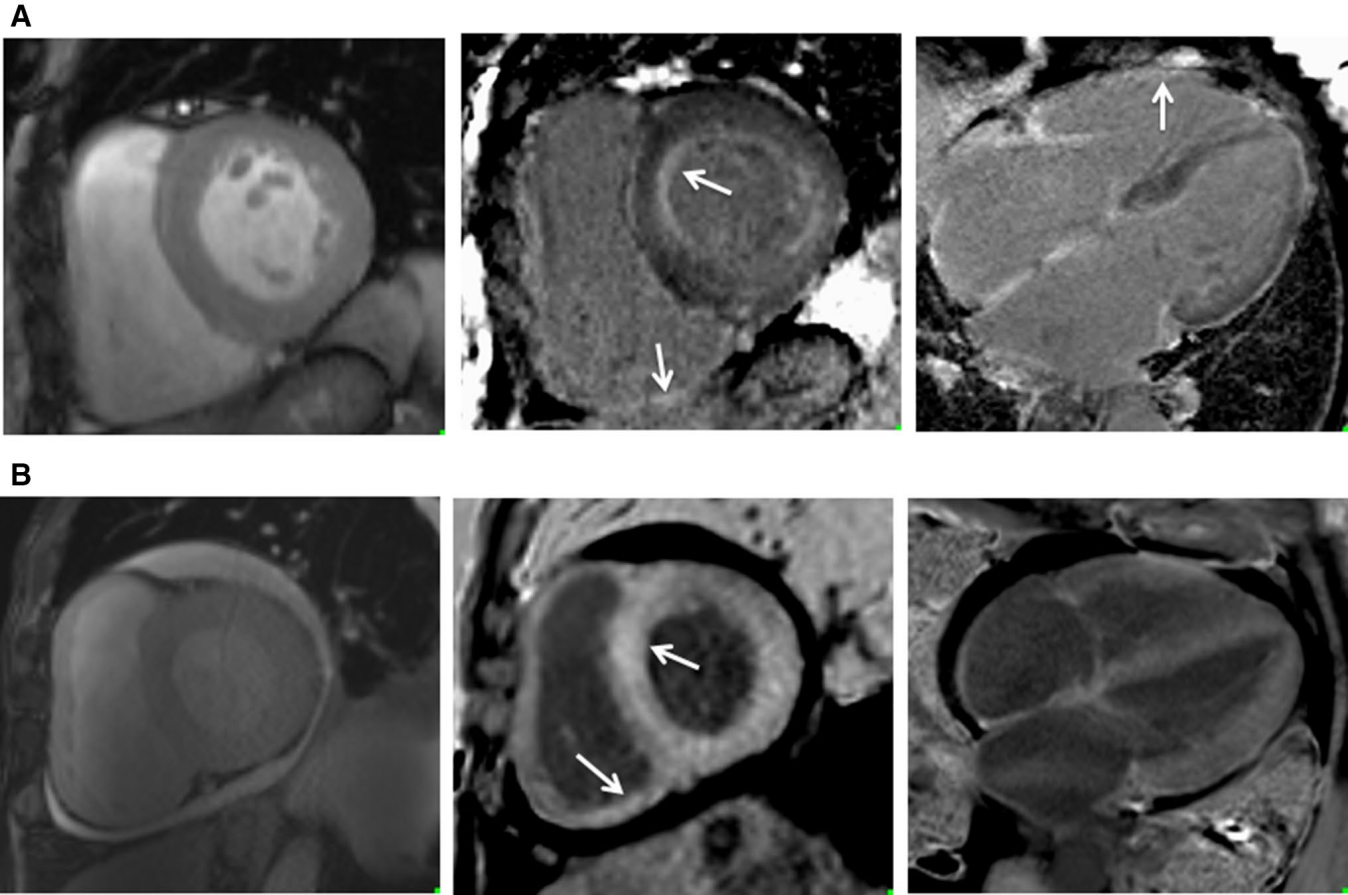
Representative two cases with cardiac amyloidosis. **a** A 68-year-old female with light chain amyloidosis. CMR cine in the short-axis view demonstrates LV hypertrophy. Cine-MR showed preserved normal biventricular systolic global LV function (LVESVi 33.5 mL/m^2^, LVEF 51.0%, RVESVi 35.5 mL/m^2^, RVEF 62.3%). The LGE-MRI images on the mid and right panel showed subendocardial LGE in the left ventricle and LGE in the right ventricle (*arrow* indicated). The patient did well during follow-up. **b** A 76-year-old male patient with reduced RVEF. CMR cine in the short-axis view demonstrates biventricular hypertrophy, increased right ventricular wall thickness, and circumferential pericardial effusion. LGE positive was shown in LV and RV (*mid*-*panel*, *arrows*). Cine-MR showed altered systolic global LV function (LVESVi 59.9 mL/m^2^, LVEF 31.0%, RVESVi 65.2 mL/m^2^, RVEF 34.0%). Despite optimal medical therapy, this patient suffered NYHA Class III heart failure and died in hospital after diagnosis with amyloidosis at the 6th month

During a mean follow-up period of 21 months, there were 27 deaths (57.4%) in the CA group. Six deaths occurred within 1 month of AL amyloidosis diagnosis, 17 within 6 months, and 24 within 1 year. No deaths occurred in patients without CA. The results of the univariate analyses in CA patients are presented in Table 3. NYHA functional class, creatinine, log NT-proBNP, LVEF, LVMI, RVEF, RVESVi, LV-LGE, and RV-LGE were significantly related to survival. Table 4 shows the results of the multivariate analyses in CA patients. RVESVi and RV-LGE were better predictors of adverse outcome than other CMR variables, including LVEF and RVEF. RVESVi (HR 1.033, 95% CI 1.004–1.063, *P* = 0.026 combined model) and RV-LGE (HR 2.814, 95% CI 1.063–7.450, *P* = 0.037 combined model) remained significantly associated with the primary outcome when all significant variables of clinical, laboratory, and CMR model were added to the model. ROC analysis revealed that the AUC of RVESVi was 0.757 (Fig. 2). The best RVESVi cut-off value for predicting death was 32 mL/m^2^, with a sensitivity of 74.1%, specificity of 74.9%, PPV of 79.9%, and NPV of 68.2%. The results of the univariate Kaplan–Meier survival analysis are shown in Fig. 3a. The cut-off of 32 mL/m^2^ discriminated the two groups with a highly significant survival difference (log rank test = 11.618, *P* < 0.001). The overall survival of the RV-LGE-negative group was superior to that of the RV-LGE-positive group (log rank test = 10.343, *P* = 0.001; Fig. 3b). Two representative cases are presented in Fig. 4a, b.

**Table 3:** Uni-Cox proportional hazard analysis of various clinical and cardiac magnetic resonance imaging predictors of long-term mortality in AL amyloid patients

	Cardiac amyloidosis		All AL amyloidosis	
	Unadjusted HR (95% CI)	*P* value	Unadjusted HR (95% CI)	*P* value
Demographics				
Age	0.989 (0.954–1.025)	0.544	1.000 (0.967–1.034)	0.999
Gender	0.702 (0.330–1.495)	0.359	0.673 (0.316–1.433)	0.305
NYHA class	1.480 (1.029–2.018)	0.035	1.903 (1.356–2.670)	<0.001
Low-voltage pattern	1.148 (0.537–2.456)	0.721	2.157 (1.007–4.623)	0.048
Biochemical biomarkers				
Creatinine	1.012 (1.001–1.023)	0.040	1.004 (1.001–1.006)	0.003
UA	1.003 (1.000–1.005)	0.063	1.016 (1.005–1.027)	0.004
AST	1.008 (0.993–1.023)	0.281	1.000 (0.988–1.012)	0.936
ALT	1.007 (0.990–1.025)	0.424	1.001 (0.986–1.018)	0.860
Log Troponin T	2.660 (0.973–7.272)	0.056	5.188 (2.126–12.660)	<0.001
Log NT-pro BNP	2.756 (1.163–6.532)	0.021	4.338 (2.090–9.004)	<0.001
CMR parameters				
LVEF	0.961 (0.930–0.994)	0.022	0.945 (0.916–0.975)	<0.001
LVEDVi	1.006 (0.987–1.025)	0.542	1.007 (0.987–1.028)	0.485
LVESVi	1.026 (1.000–1.054)	0.051	1.038 (1.012–1.065)	0.004
LVMI	1.012 (1.001–1.023)	0.029	1.018 (1.008–1.029)	0.001
RVEF	0.957 (0.929–0.984)	0.002	0.941 (0.915–0.969)	<0.001
RVEDVi	1.012 (0.994–1.031)	0.193	1.007 (0.988–1.027)	0.475
RVESVi	1.046 (1.019–1.074)	0.001	1.059 (1.031–1.087)	<0.001
LV-LGE	8.326 (1.126–61.562)	0.038	23.222 (3.139–171.783)	0.002
RV-LGE	3.010 (1.341–6.758)	0.008	7.101 (2.839–17.759)	<0.001

**Table 4:** Multivariate models predicting mortality in cardiac amyloidosis

Cox regression	Wald Chi square	Hazard ratio	95% CI	*P* value
Simple models				
Model 1: clinical model				
NYHA class	4.63	1.480	1.029–2.128	0.035
Model 2: biochemical model				
Log NT-pro BNP	4.89	2.646	1.117–6.270	0.027
Model 3: CMR model				
RVESVi	11.86	1.033	1.004–1.063	0.026
RV-LGE	16.17	2.814	1.063–7.450	0.037
Combined models [models 1 + 2+3 (all significant variable model 1–2–3)]				
RVESVi	11.86	1.033	1.004–1.063	0.026
RV-LGE	16.17	2.814	1.063–7.450	0.037

### Univariate and multivariate analyses in all AL patients

The unadjusted variables associated with all-cause death analysis in patients with AL amyloidosis (including both CA and non-CA patients) are reported in Table 3. By multivariate analysis, only log NT-proBNP and RV-LGE exhibited a statistically significant association with mortality (HR 3.412, 95% CI 1.484–7.845, *P* = 0.004 and HR 4.149, 95% CI 1.623–10.607, *P* = 0.003, respectively) after adjustment for the significant variables of the clinical, biological, and CMR model (Table 5).

**Table 5:** Multivariate models predicting mortality in AL amyloidosis

Cox regression	Wald Chi square	Hazard ratio	95% CI	*P* value
Simple models				
Model 1: clinical model				
NYHA class	13.867	1.480	1.029–2.128	<0.001
Model 2: biochemical model				
Log NT-pro BNP	15.504	4.338	2.090–9.004	<0.001
Model 3: CMR model				
RVEF	4.619	0.967	0.939–0.997	0.032
LV-LGE	3.629	8.338	0.941–73.896	0.057
RV-LGE	3.069	2.411	0.901–6.456	0.080
Combined models [models 1 + 2+3 (all significant variables model 1–2–3)]				
Log NT-pro BNP	8.345	3.412	1.484–7.845	0.004
RV-LGE	8.829	4.149	1.623–10.607	0.003

## Discussion

Cardiac involvement is common in patients with AL amyloidosis and is characterized by a poor prognosis with limited treatment options [16]. In the present study, our results indicate that higher RVESVi and positive RV-LGE portend a worse prognosis in patients with AL CA. In patients with AL amyloidosis, including both CA patients and non-CA patients, NT-proBNP and RV-LGE were independent predictors of death.

### Prognostic value of RV-LGE

LGE CMR is a robust technique used to assess myocardial irreversible injury, such as myocardial focal fibrosis [17] and amyloidosis [18]. Thus, the use of LGE CMR imaging has recently become a routine examination for the evaluation of amyloid patients for the presence of LGE. A previous study showed that LGE was a strong predictor of 1-year mortality in patients with suspected CA [13]. A recent study described transmural LGE as an important marker of all-cause mortality in systemic amyloidosis [14]. Banypersad and colleagues [19] proposed that extracellular volume fraction measured by T1 mapping used as a predictor of amyloid burden had a strong predictive role in patients with AL amyloidosis. However, one of the known limitations of T1 mapping is the sequence- and vendor-specific difference, which limits the role of T1 mapping in routine clinical practice. The LGE technique-based PSIR reconstruction makes LGE a reliable prognostic parameter in patients with cardiac amyloidosis. Multivariate analysis revealed a 2.8-fold higher risk of death when RV-LGE was present in our cohort. This data indicated that the visual recognition of RV-LGE is a strong and independent predictor of patient mortality. In a recent study by Bodez et al. [9], tricuspid annular plane systolic excursion (TAPSE) was a significant independent predictor of mortality (HR 1.08, 95% CI 1.01–1.15), whereas RV-LGE was not significant. We did not include TAPSE in our analysis and the differential importance of RV-LGE might also be due to different patient population studied (transthyretin-related CA in theirs versus AL-related CA in our study).

### Prognostic value of RV functional parameter

As LV ejection fraction is generally preserved in patients with CA, other markers of myocardial dysfunction are needed for prognosis. Previous echocardiographic studies have shown that changes in RV function have independent prognostic power in patients with amyloidosis, such as Tei index ≥0.9 [20], TAPSE <17 mm [8], and peak longitudinal systolic basal anteroseptal strain ≤−7.5% [5]. CMR is the gold-standard to evaluate RV function and there are limited data on CMR RV parameters for prognosis in CA patients. Our data demonstrate a decrease of biventricular function between survivors and non-survivors at 6-month follow-up and we have found that an increased RVESVi carried a negative prognosis.

Previous echocardiographic studies have demonstrated that changes in LV function play a role in predicting mortality, such as mitral inflow deceleration time [21] and *E*/*A* and *E*/*E′* ratios [3]. An echocardiographic finding of shorter LV ejection time (240 ms) has independent and additive prognostic value to clinical heart failure evaluation in determining long-term survival of AL patients [22]. We found LVEF not to be a marker of all-cause mortality in multivariate analysis after adjustment for established prognostic indicators, including LV-LGE, RV-LGE, RV parameters, LVMI, and NYHA functional class, but we have not evaluated the prognostic power of diastolic parameters of the LV filling as shown by the echocardiographic studies. In addition, our study identified the value of NT-proBNP and RV-LGE as independent factors of death in all amyloid patients. This result is consistent with the Mayo Clinic staging system that shows that troponin T and NT-proBNP are independently prognostic for overall survival in patients with AL amyloidosis [23].

### Clinical implications

We investigated the prognostic value of RV volume, function, and tissue characteristics using CMR in AL patients with CA. On multivariate analysis, RVESVi and RV-LGE are independently associated with an adverse outcome. Furthermore, in patients with CA, an RVESVi greater than 32 mL/m^2^ identified a cohort of patients who may be at high risk of death. There is some variability in survival and clinical course in AL patients, and it is important to be able to stratify these patients to guide therapeutic decisions. Prospective trials aimed at early detection of poor prognosis and employment of aggressive treatment strategies such as transplantation or chemotherapy with stem cell transplantation should be conducted to provide therapeutic guidance.

### Study limitations

The present study has several limitations. The patient sample size was relatively small; however, given the low prevalence of CA, our study numbers compare favorably with previously published studies. Endomyocardial biopsy was not performed, but AL amyloidosis was found in other organs, and all patients met the criteria for clinical diagnosis of CA. Diagnostic algorithms integrating clinical presentation, electrocardiography, echocardiography, and blood biomarkers can obviate the need for myocardial biopsy. Furthermore, cardiac response assessment is not regularly performed after chemotherapy at our center. Finally, our study was performed in a tertiary referral center, and our results may not be extrapolated to all AL populations.

## Conclusions

RV size and tissue characteristics are significantly abnormal in patients with cardiac amyloid, in which the accurate assessment of RV by CMR provides significant independent predicting value for mortality in patients with amyloidosis.

## References

[1] Merlini G, Bellotti V (2003). Molecular mechanisms of amyloidosis. N Engl J Med.

[2] Rapezzi C, Merlini G, Quarta CC, Riva L, Longhi S, Leone O, Salvi F, Ciliberti P, Pastorelli F, Biagini E, Coccolo F, Cooke RM, Bacchi-Reggiani L, Sangiorgi D, Ferlini A, Cavo M, Zamagni E, Fonte ML, Palladini G, Salinaro F, Musca F, Obici L, Branzi A, Perlini S (2009). Systemic cardiac amyloidoses: disease profiles and clinical courses of the 3 main types. Circulation.

[3] Klein AL, Hatle LK, Taliercio CP, Taylor CL, Kyle RA, Bailey KR, Seward JB, Tajik AJ (1990). Serial Doppler echocardiographic follow-up of left ventricular diastolic function in cardiac amyloidosis. J Am Coll Cardiol.

[4] Koyama J, Ray-Sequin PA, Falk RH (2003). Longitudinal myocardial function assessed by tissue velocity, strain, and strain rate tissue Doppler echocardiography in patients with AL (primary) cardiac amyloidosis. Circulation.

[5] Bellavia D, Pellikka PA, Al-Zahrani GB, Abraham TP, Dispenzieri A, Miyazaki C, Lacy M, Scott CG, Oh JK, Miller FA (2010). Independent predictors of survival in primary systemic (Al) amyloidosis, including cardiac biomarkers and left ventricular strain imaging: an observational cohort study. J Am Soc Echocardiogr.

[6] Yamamura S, Izumiya Y, Ishida T, Onoue Y, Kimura Y, Hanatani S, Araki S, Fujisue K, Sueta D, Kanazawa H, Takashio S, Usuku H, Sugamura K, Sakamoto K, Yamamoto E, Yamamuro M, Yasuda H, Kojima S, Kaikita K, Hokimoto S, Ogawa H, Tsujita K (2016). Reduced trans-mitral A-wave velocity predicts the presence of wild-type transthyretin amyloidosis in elderly patients with left ventricular hypertrophy. Heart Vessel.

[7] Cappelli F, Porciani MC, Bergesio F, Perlini S, Attana P, Moggi Pignone A, Salinaro F, Musca F, Padeletti L, Perfetto F (2012). Right ventricular function in AL amyloidosis: characteristics and prognostic implication. Eur Heart J Cardiovasc Imaging.

[8] Ghio S, Perlini S, Palladini G, Marsan NA, Faggiano G, Vezzoli M, Klersy C, Campana C, Merlini G, Tavazzi L (2007). Importance of the echocardiographic evaluation of right ventricular function in patients with AL amyloidosis. Eur J Heart Fail.

[9] Bodez D, Ternacle J, Guellich A, Galat A, Lim P, Radu C, Guendouz S, Bergoend E, Couetil JP, Hittinger L, Dubois-Rande JL, Plante-Bordeneuve V, Deux JF, Mohty D, Damy T (2016). Prognostic value of right ventricular systolic function in cardiac amyloidosis. Amyloid.

[10] Champion HC, Michelakis ED, Hassoun PM (2009). Comprehensive invasive and noninvasive approach to the right ventricle-pulmonary circulation unit: state of the art and clinical and research implications. Circulation.

[11] Vogelsberg H, Mahrholdt H, Deluigi CC, Yilmaz A, Kispert EM, Greulich S, Klingel K, Kandolf R, Sechtem U (2008). Cardiovascular magnetic resonance in clinically suspected cardiac amyloidosis: noninvasive imaging compared to endomyocardial biopsy. J Am Coll Cardiol.

[12] Migrino RQ, Christenson R, Szabo A, Bright M, Truran S, Hari P (2009). Prognostic implication of late gadolinium enhancement on cardiac MRI in light chain (AL) amyloidosis on long term follow up. BMC Med Phys.

[13] Austin BA, Tang WH, Rodriguez ER, Tan C, Flamm SD, Taylor DO, Starling RC, Desai MY (2009). Delayed hyper-enhancement magnetic resonance imaging provides incremental diagnostic and prognostic utility in suspected cardiac amyloidosis. JACC Cardiovasc Imaging.

[14] Fontana M, Pica S, Reant P, Abdel-Gadir A, Treibel TA, Banypersad SM, Maestrini V, Barcella W, Rosmini S, Bulluck H, Sayed RH, Patel K, Mamhood S, Bucciarelli-Ducci C, Whelan CJ, Herrey AS, Lachmann HJ, Wechalekar AD, Manisty CH, Schelbert EB, Kellman P, Gillmore JD, Hawkins PN, Moon JC (2015). Prognostic value of late gadolinium enhancement cardiovascular magnetic resonance in cardiac amyloidosis. Circulation.

[15] Comenzo RL, Reece D, Palladini G, Seldin D, Sanchorawala V, Landau H, Falk R, Wells K, Solomon A, Wechalekar A, Zonder J, Dispenzieri A, Gertz M, Streicher H, Skinner M, Kyle RA, Merlini G (2012). Consensus guidelines for the conduct and reporting of clinical trials in systemic light-chain amyloidosis. Leukemia.

[16] Kyle RA, Gertz MA (1995). Primary systemic amyloidosis: clinical and laboratory features in 474 cases. Semin Hematol.

[17] Korkusuz H, Esters P, Huebner F, Bug R, Ackermann H, Vogl TJ (2010). Accuracy of cardiovascular magnetic resonance in myocarditis: comparison of MR and histological findings in an animal model. J Cardiovasc Magn Reson.

[18] Hashimura H, Ishibashi-Ueda H, Yonemoto Y, Ohta-Ogo K, Matsuyama TA, Ikeda Y, Morita Y, Yamada N, Yasui H, Naito H (2016). Late gadolinium enhancement in cardiac amyloidosis: attributable both to interstitial amyloid deposition and subendocardial fibrosis caused by ischemia. Heart Vessel.

[19] Karamitsos TD, Piechnik SK, Banypersad SM, Fontana M, Ntusi NB, Ferreira VM, Whelan CJ, Myerson SG, Robson MD, Hawkins PN, Neubauer S, Moon JC (2013). Noncontrast T1 mapping for the diagnosis of cardiac amyloidosis. JACC Cardiovasc Imaging.

[20] Liu D, Hu K, Herrmann S, Cikes M, Ertl G, Weidemann F, Stork S, Nordbeck P (2017). Value of tissue Doppler-derived Tei index and two-dimensional speckle tracking imaging derived longitudinal strain on predicting outcome of patients with light-chain cardiac amyloidosis. Int J Cardiovasc Imaging.

[21] Austin BA, Duffy B, Tan C, Rodriguez ER, Starling RC, Desai MY (2009). Comparison of functional status, electrocardiographic, and echocardiographic parameters to mortality in endomyocardial-biopsy proven cardiac amyloidosis. Am J Cardiol.

[22] Migrino RQ, Harmann L, Christenson R, Hari P (2014). Clinical and imaging predictors of 1-year and long-term mortality in light chain (AL) amyloidosis: a 5-year follow-up study. Heart Vessel.

[23] Kumar S, Dispenzieri A, Lacy MQ, Hayman SR, Buadi FK, Colby C, Laumann K, Zeldenrust SR, Leung N, Dingli D, Greipp PR, Lust JA, Russell SJ, Kyle RA, Rajkumar SV, Gertz MA (2012). Revised prognostic staging system for light chain amyloidosis incorporating cardiac biomarkers and serum free light chain measurements. J Clin Oncol.

